# The Genome of the Lima Bean Variety Baiyu Bean Highlights Its Evolutionary Characteristics

**DOI:** 10.1002/ece3.71027

**Published:** 2025-02-28

**Authors:** Fengqi Li, Junfang Liu, Youssef Dewer, Muhammad Haseeb Ahsan, Chunyan Wu

**Affiliations:** ^1^ State Key Laboratory of Green Pesticide, Key Laboratory of Green Pesticide and Agricultural Bioengineering, Ministry of Education Center for R&D of Fine Chemicals of Guizhou University Guiyang China; ^2^ Nanyang Institute of Technology Nanyang China; ^3^ Phytotoxicity Research Department, Central Agricultural Pesticide Laboratory Agricultural Research Center Giza Egypt; ^4^ College of Plant Protection Yangzhou University Yangzhou China

**Keywords:** baiyu bean, genome, molecular evolution, structural variation

## Abstract

The baiyu bean ( *Phaseolus lunatus* ), also known as the lima bean, is a plant belonging to the Fabaceae family, has a long and distinguished history of cultivation in China and is a highly regarded local variety of lima bean. In the current study, we present the reference genome of the baiyu bean variety, which has a scaffold N50 length of 47.545 Mb. A comparative genomic analysis was conducted using genomes of seven legume species, and the results demonstrated that 1564 and 1275 genes of baiyu bean exhibited expansion and contraction, respectively. Moreover, 543 genes were identified as exclusive to the baiyu bean. The analysis of adaptive evolution genes revealed the presence of 61 genes under adaptive evolution between *P. lunatus* and the common bean *P. vulgaris* . An examination of the branch model revealed the presence of five genes undergoing adaptive evolution in the *P. lunatus* branch. Additionally, the evolutionary selective pressure acting on other branches of legume plants was analyzed. A comprehensive analysis of structural variations (SVs) between the baiyu bean and G27455 genome was conducted, resulting in the identification of 5549 SVs. Among these, 333 genes were identified as high‐impact SV genes. The acquisition of the genome sequence of this excellent variety will facilitate the exploration and utilization of its characteristics, providing a foundation for the genetic improvement of the lima bean.

## Graphical abstract

The reference genome of the baiyu bean was conducted, which has a contig N50 length of 47.55 Mb. Through comparative genomic analysis of legume species, gene families that have expanded and contracted in the baiyu bean, species‐specific genes, and genes under adaptive evolution have been identified. A total of 5549 structural variations (SVs) between the G27455 genome and the baiyu bean were found. Among them, 333 genes were found to be SV‐high‐impact genes.

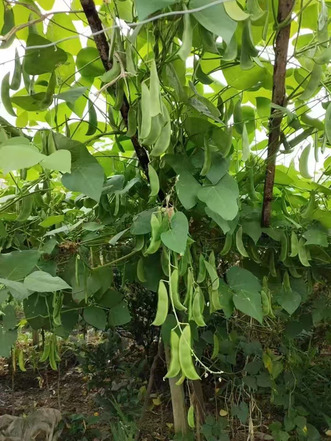

## 1 Introduction

Lima bean ( *Phaseolus lunatus* L.), common bean ( *P. vulgaris* L.), ayocote or runner bean ( *P. coccineus* L.), tepary bean ( *P. acutifolius* A. Grey), and *P. dumosus* year‐long bean ( *P. dumosus* Macfady) are the five domesticated species among the approximately 70 species of the *Phaseolus* genus that have been reported (Bitocchi et al. 2017; Garcia et al. 2021). In terms of agronomy and economics, lima beans and common beans are the most significant species in the *Phaseolus* genus, which are globally cultivated. Lima beans are a nutrient‐dense food because of their low glycemic index, high fiber, protein, and slow‐release carbohydrate content (Bello‐Pérez et al. 2007).

Baiyu bean is a local variety of lima bean in China, mainly cultivated in Jiangxi, Zhejiang, Fujian, and other provinces in the lower and middle reaches of the Yangtze River. This variety has a cultivation history of approximately 300 years in Shangrao and Jiangxi Provinces. Baiyu bean seeds contain at least 20% protein and > 50% carbohydrate. It also contains various amino acids, including isoleucine, valine, phenylalanine, lysine, leucine, and methionine. Additionally, this variety has excellent characteristics, such as resistance to diseases and pests (Xu and Shen 2020).

Genome sequencing technology provides a complete genetic resource and is a powerful tool for analyzing the genetic characteristics of superior varieties, natural product biosynthetic pathways, and molecular evolutionary analysis. Legumes, including both warm‐ and cool‐season varieties, are essential in agriculture because of their nitrogen‐fixing ability, enriching soil fertility, and offering protein‐rich food and forage (Graham and Vance 2003). Warm‐season legumes, such as lima beans, common beans, and soybeans, thrive in hot climates and are planted in late spring or summer, making them ideal for tropical and subtropical regions (Yamaguchi 1983). Among warm‐season legumes, the lima bean thrives in hotter and drier environments that are less suitable for the growth of the common bean (Maquet et al. 1999). The adaptive evolution of plants is closely tied to temperature, as it influences physiological processes, metabolic rates, and ecological fitness, driving species to develop traits suited to specific thermal environments (Yamori et al. 2014). The ratio of nonsynonymous substitutions (ka) to synonymous substitutions (ks), denoted by ω (ka/ks), is a common metric for detecting adaptive evolution (Hurst 2002; Yang and Bielawski 2000). Typically, ω > 1 signifies positive selection, ω < 1 reflects purifying selection, and ω = 1 indicates neutral evolution. The physiological traits of warm‐season legumes and lima beans may be driven by adaptive evolution, but the specific genes and signaling pathways involved remain unclear.

In this study, we present a high‐quality genome of the baiyu bean variety and analyze the evolutionary properties of the genome of this variety. To obtain this high‐quality genome, we further utilized genomic data to conduct comparative genomic analysis between lima beans and other leguminous plants, as well as between the baiyu bean and G27455. Through systematic comparative genomic analysis, we aimed to reveal the molecular evolutionary characteristics of lima beans and this cultivar.

## 2 Materials and Methods

**FIGURE 1:**
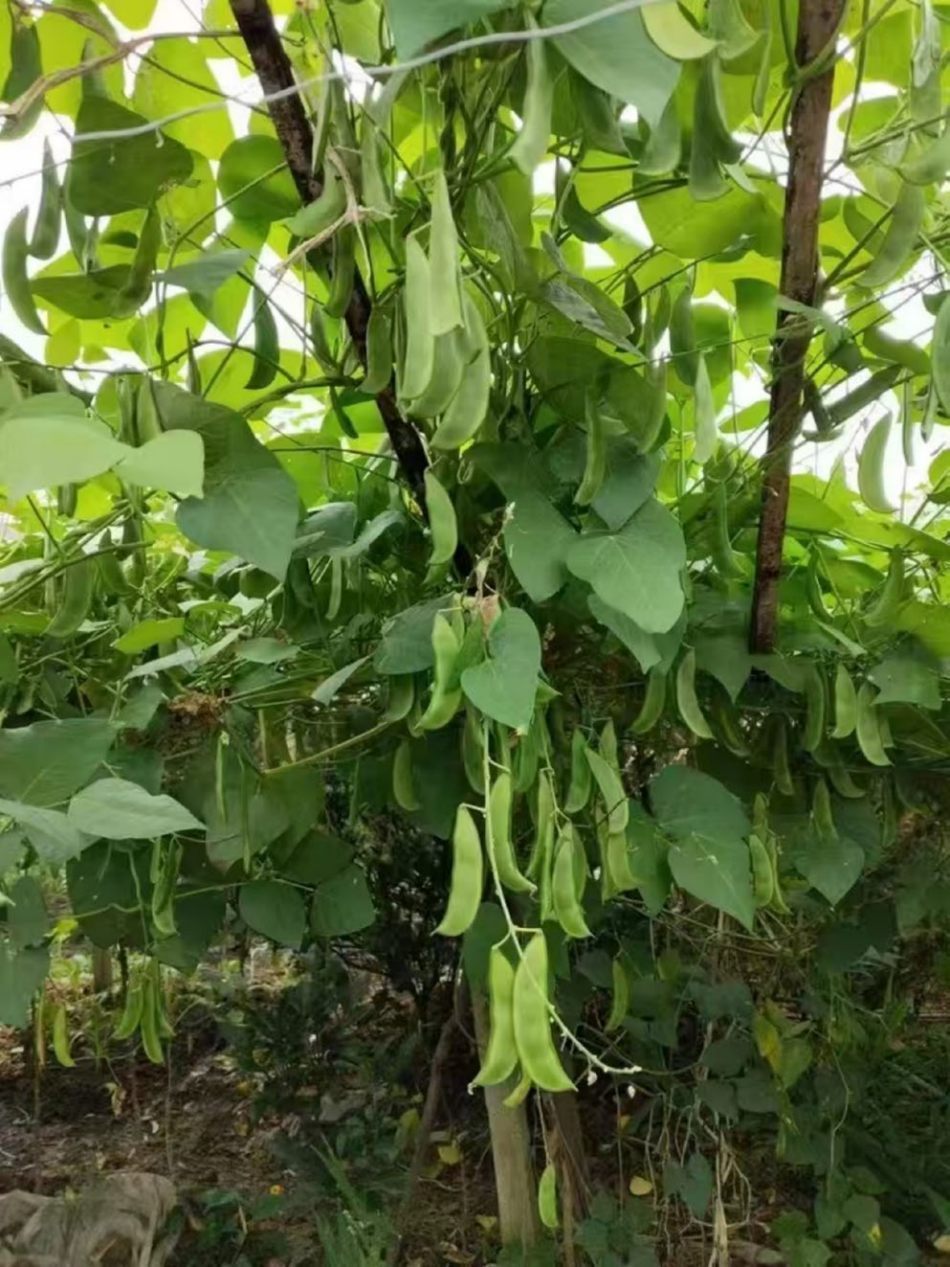
Baiyu bean plants cultivated in the field.

Baiyu bean plants cultivated in the fields of Yushan, Shangrao City, Jiangxi Province, China (Figure 1), were used in this study. After harvest, the seeds were germinated indoors and subsequently grown in a greenhouse maintained at 26°C with a photoperiod of 16 h light and 8 h dark (LD:SD = 16:8). Young leaf tissue samples were collected at the 10‐day seedling stage and preserved in liquid nitrogen for subsequent DNA extraction. Genomic DNA was extracted using the cetyltrimethylammonium bromide (CTAB) method (Aboul‐Maaty and Oraby 2019). DNA quality and concentration were assessed by 1% agarose gel electrophoresis and a Qubit fluorimeter (Invitrogen, Carlsbad, CA). High‐quality DNA was used for subsequent sequencing using the Nanopore, Illumina, and Hi‐C methods.

### 2.1 Library Construction and Genome Sequencing

Short‐read libraries were constructed according to the manufacturer's guidelines (Illumina, San Diego, CA) and subsequently sequenced using the Illumina NovaSeq 6000 platform. Illumina sequencing libraries were prepared for long‐read DNA sequencing to assess genome size, refine genome assembly, and evaluate assembly quality. Paired‐end libraries with insert sizes of 300 bp were constructed according to the Illumina standard protocol and sequenced using paired‐end (2 × 150 bp) on a Novaseq6000 sequencer (Illumina Inc., San Diego, CA, USA). After eliminating reads with low‐quality bases, adapter sequences, and duplicates using fastp (Chen et al. 2018), the resulting clean reads were used for subsequent analysis. A total of 59.06 Gb of clean data were obtained, resulting in approximately 54× genome coverage. The frequency of 17‐nucleotide k‐mers was used for genome survey analysis.

To produce Oxford Nanopore long reads, 15 μg of genomic DNA was size‐selected in high‐pass mode (> 20 kb) using the BluePippin BLF7510 cassette (Sage Science), and it was processed using the standard ligation sequencing kit SQK‐LSK109 from Oxford Nanopore Technologies. Sequencing was performed using FLO‐MIN106 (R9.4.1) flow cells on the PromethION beta platform. Base calling of raw signal data was facilitated using Guppy version 6.1.2. Reads that met the quality threshold (Q > 10) were processed to remove contaminants using Centrifuge (v1.0.4). A minimum match length of 50 bp was required for alignment against the NCBI RefSeq databases for bacteria, archaea, and fungi (Kim et al. 2016).

Leaf samples from seedlings of the same plant were used for Hi‐C sequencing. Then, chromatin extraction, digestion, DNA ligation, purification, and fragmentation were performed to create Hi‐C libraries (Belton et al. 2012). Hi‐C sequencing was performed on the Illumina NovaSeq 6000 platform (San Diego, CA, USA). Trimmomatic version 0.36 (Bolger et al. 2014) was used to process Illumina reads from the Hi‐C collection to remove adapter and low‐quality sequences. For further study, 59.83 GB of clean data were retained.

Roots, leaves, and stems were collected from three individual baiyu bean plants for RNA preparation in the context of RNA‐seq. RNA integrity was assessed using the RNA Nano 6000 Assay Kit with a Bioanalyzer 2100 system (Agilent Technologies, Santa Clara, CA, USA). rRNA was removed from total RNA using the Ribo‐Zero rRNA Removal Kits (Plant) following the manufacturer's instructions (Illumina, San Diego, CA, USA). Total RNA was used to remove rRNA, and the quality of the RNA was evaluated using an Agilent 2100 Bioanalyzer. Strand‐specific RNA libraries were generated using AHTS Universal KAPA RNA HyperPrep Kits for Illumina, followed by 150 bp paired‐end sequencing on a Novaseq 6000 (Illumina, USA). The raw RNA sequencing data for lima bean treatments involving *Tetranychus cinnabarinus* and Alamethicin were sourced from our prior research (Li et al. 2015, 2018).

### 2.2 De Novo Assembly of the Baiyu Bean Genome

NextDenovo version 2.3.0 was employed for genome assembly, encompassing sequencing error correction, preliminary assembly, and genome polishing. The NextCorrect module was employed for the correction of raw reads and extraction of consensus sequences. The NextGraph module enabled initial assembly, whereas the NextPolish module was employed for genome refinement (Hu et al. 2020). In the genome polishing stage, nanopore reads were employed three times, whereas Illumina sequencing reads were used four times for genome correction. The seed cutoff was set at 30 kb, and the read cutoff was set at 1 kb for the NextDenovo genome assembly, with default parameters applied to other settings. The assembly was conducted using quality‐controlled reads in NextDenovo. The preliminary assembly was refined using Nextpolish v. 1.2.4. At this stage, Nanopore long reads and Illumina short reads were used three times for genome correction.

The cleaned Hi‐C reads were set to the assembled contigs using BWA v. 0.7.1753 (Li and Durbin 2009). Contigs were clustered into scaffolds using SALSA (v2.2) based on the alignments, with parameters set to ‘‐e GATC ‐i 3’ (Ghurye et al. 2019). Juicebox Assembly Tools manually correct false scaffolding joins. In addition, the homology‐based scaffolds were generated with RagTag v2.0.1 (Alonge et al. 2019) “scaffold,” using the G27455 genome assembly (Garcia et al. 2021) as a reference. Then, the Hi‐C‐based scaffolds and homology‐based scaffolds were reconciled with RagTag “merge” according to the method recently described by Alonge et al. (2019). Finally, the scaffolds were manually corrected using Juicebox Assembly Tools v1.9.9. The final assembly was examined for off‐target contaminants using the NCBI‐FCS‐GX v0.5.4 tool (Astashyn et al. 2024). BUSCO v. 5.1.2 (Manni et al. 2021), with the viridiplantae_odb10 database, was used to evaluate the completeness and accuracy of the assembled genome.

### 2.3 Genome Annotation

A previously reported dataset of common bean repeats (Schmutz et al. 2014) was utilized to mask identified transposable elements in the baiyu bean genome using RepeatMasker (v.4.1.0, http://www.repeatmasker.org). After masking the repeat elements, gene structures were predicted using a combination of transcriptome‐based, ab initio, and homology‐based approaches. The final gene set was assessed utilizing BUSCO v5.1.2 (Manni et al. 2021). The functions of the baiyu bean genes were annotated utilizing eggNOG‐mapper v2.1.12 in conjunction with the orthologous gene database (Cantalapiedra et al. 2021).

### 2.4 Genome Evolution Analysis

For phylogenetic analysis, the protein sequences of the baiyu bean and six additional legume species were employed, with *Arabidopsis thaliana* (Arabidopsis Genome Initiative 2000) serving as an outgroup. The list includes *Cicer arietinum* , *Glycine max* , *Lotus japonicus*, *Cajanus cajan* , *Medicago truncatula* , and *Phaseolus vulgaris* . Genomic data were obtained from the Phytozome V13 website at https://phytozome‐next.jgi.doe.gov. Only the longest transcript from each gene was used for further analysis. By using OrthoFinder, version 3.0.1.b1, orthologous genes were identified (Emms and Kelly 2019). The phylogenetic tree was reconstructed using 642 single‐copy orthologs derived from the aforementioned results. The species tree was generated using IQ‐tree v1.6.12 (Minh et al. 2020). The resulting phylogenetic tree is represented using FigTree. The MCMCTREE program in PAML v4.9j (Yang 2007) (http://abacus.gene.ucl.ac.uk/software/paml.html) was used to estimate divergence periods among these species. Three fossil records from the TimeTree database were used as calibration points (http://www.timetree.org). These dates correspond to the *G. max–P. vulgaris* divergence (19.5–28.8 Ma) and the *A. thaliana–L. japonicus* divergence (102.0–112.5 Ma) respectively. Using the CAFÉ v4.1 tool (Han et al. 2013), we performed expansion and contraction analysis. Based on ‘all‐versus‐all’ BLASTp (*E*‐value 1e−5) alignments, MCscanX 2 (Wang et al. 2012) was used to detect syntenic blocks within the baiyu bean and between the baiyu bean and the common bean. Circos software was used to display synteny blocks in the baiyu bean (Krzywinski et al. 2009). The 4DTv values were computed according to earlier instructions (Hasegawa et al. 1985).

The positive and negative selection between *P. vulgaris* and *P. lunatus* was assessed using the Ka/Ks ratio. The Ka/Ks ratio for each orthologous gene pair was calculated using the YN method in KaKs_Calculator 3.0 software (Zhang 2022). Using *P. lunatus* , *P. vulgaris* , *G. max* , and *C. cajan* as the foreground branches (Ahmed et al. 2022), PosiGene v0.1 (Sahm et al. 2017) was employed for whole‐genome detection of positively selected genes (PSGs). We identified the PSGs in these branches and conducted functional enrichment analysis for these PSGs utilizing a *p* value threshold of 0.05 following false discovery rate (FDR) correction. KOBAS‐i was used to analyze the GO and KEGG enrichment of orthologous gene pairs exhibiting Ka/Ks ratios exceeding 1 (Bu et al. 2021). To avoid false‐positive results owing to recombination events, we employed PhiPack to detect recombination events (Bruen et al. 2006). Using the three statistical tests provided in PhiPack (Phi, NSS, and MaxChi2), we assessed the occurrence of recombination. Recombination was considered to have occurred if at least two of these three models indicated recombination events.

### 2.5 Genomic SVs Between G27455 and Baiyu Bean

Three different approaches were used to compare the structural variants (SVs) of G27455 and the baiyu bean. One approach involved utilizing Assemblytics version 1.2.1 (Nattestad and Schatz 2016) to align genome assemblies and identify SVs. Two additional nanopore reads were mapped to the G27455 reference genome using NGMLR (v0.2.7) (Sedlazeck et al. 2018). Subsequently, Sniffles (v1.0.12) (Sedlazeck et al. 2018) and SVIM (v2.0.0) (Heller and Vingron 2019) were used for SV calling. Then, using SURVIVOR version 1.0.7, the three approaches mentioned above were combined to produce consensus SVs (Jeffares et al. 2017). SnpEff 5.2c was used to annotate SVs (Cingolani et al. 2012). GO and KEGG enrichment analyses of high‐impact genes were conducted on the KOBAS‐i web server (Bu et al. 2021).

## 3 Result

### 3.1 Sequencing and Assembly of the Baiyu Bean Genome

**FIGURE 2:**
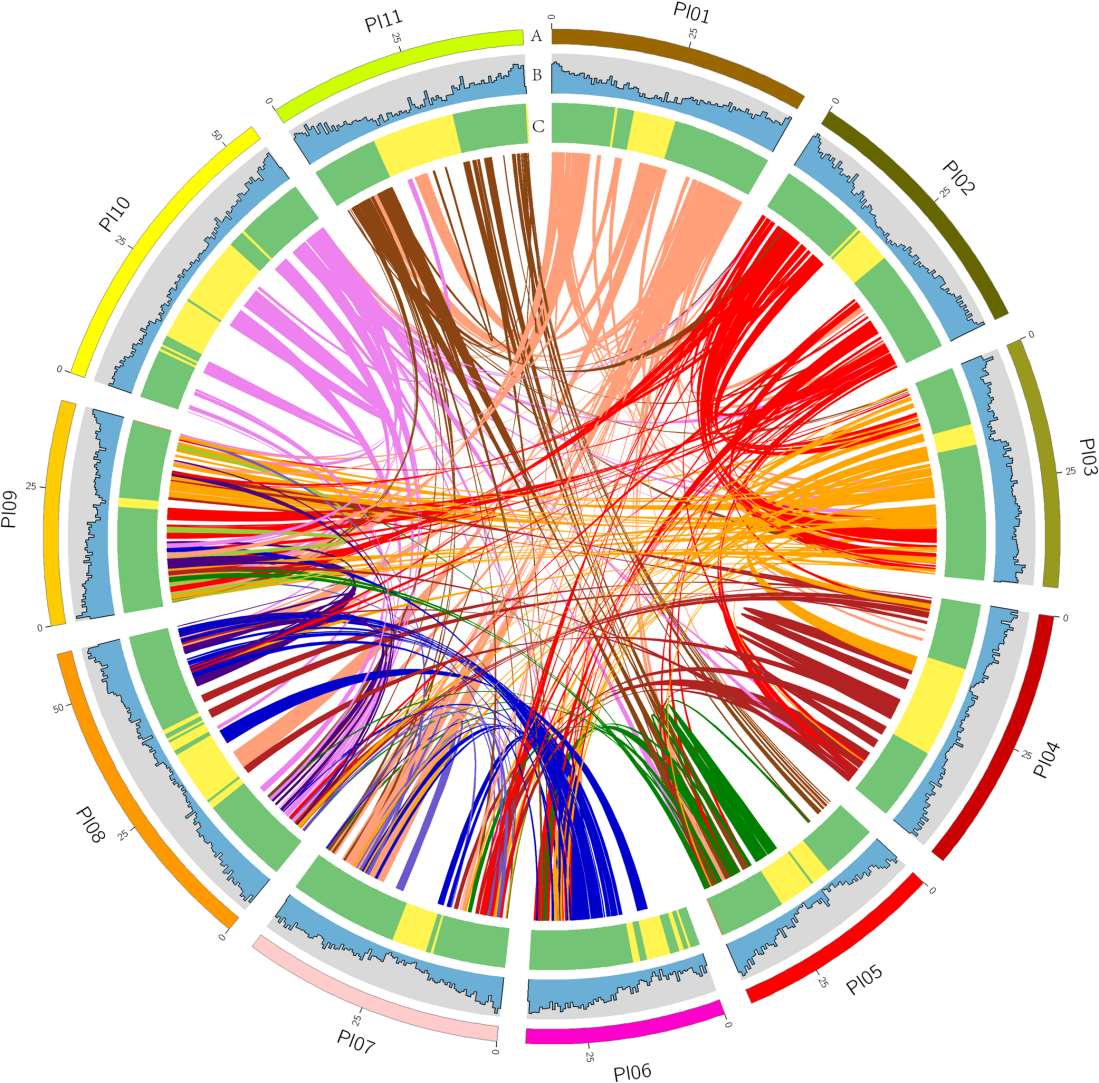
Distribution of basic genomic elements in baiyu bean. (A) Pseudomolecules. (B) Gene density (mRNA): Frequency of sites within gene regions per 500 kb. (C) GC content: Ratio of GC sites per 500 kb. Inner ribbons indicate self‐collinearity of baiyu bean, and the homologous regions of more than 1 Mb are highlighted. Circos software was used to construct the diagram.

Chromosome‐scale assembly and whole‐genome sequencing were performed on the lima bean cultivar ‘baiyu bean’. The generated sequence produced approximately 131‐fold nanopore long‐read data (71.85 Gb with a N50 read length of 26.98 kb), 130‐fold Illumina short reads (71.13 Gb), and 113‐fold Hi‐C data (59.83 Gb) following the removal of reads that were low quality. The nanopore data assembly utilizing Next Denovo resulted in a genome size of 547.93 Mb, featuring the longest contig of 24.79 Mb and contig N50 of 6.62 Mb. The initial contigs were polished using Illumina short and long nanopore reads. A total of 199.44 million read pairs were generated using Hi‐C scaffolding. Following the mapping of Hi‐C reads to the NextDenovo polished assembly, a total of 132.68 million valid interaction pairs, representing 66.52% of the unique mapped read pairs, were utilized for the Hi‐C analysis. The final assembled contigs were grouped into 11 pseudomolecules using synergistically combined homology‐ and Hi‐C‐based scaffolding solutions (Figure 2 and Table S1). The pseudochromosomes range in size from 36.37 Mb (Pl06) to 59.33 Mb (Pl08) (Table S1). The statistics for the baiyu bean genome assembly are shown in Table 1. The scaffold N50 and contigs N50 of the final assembly were 47.545 Mb and 5.479 Mb, respectively (Table 1). According to BUSCO analysis, the baiyu bean assembly contained approximately 99.1% of the core conserved plant genes (Table S2). Furthermore, a high degree of genome coverage was supported by the fact that 96.24% of transcriptome data could be mapped back to the assembly.

**TABLE 1:** Assembly statistics of the baiyu bean genome.

Genome size	546 Mb
GC content %	35.83
Scaffolds number	225
N50 length (scaffolds)	47.545 Mb
Contigs number	537
N50 length (contigs)	5.479 Mb
Longest contig	59,330,081 bp
Gene number	39,375
Chromosomes	12
Repeat	0.4111
Average gene length	2930 bp
Exons per gene	4.7

Following repeated annotation, 41.11% of the genome was identified as repetitive, with long terminal repeats (LTRs) comprising the majority (32.36%) of repeat sequences (Table S3). Homology‐based, de novo, and RNA‐seq methods were used to predict protein‐coding genes. The baiyu bean genome is estimated to contain 39,375 gene models that encode a total of 39,421 proteins following the masking of repetitive elements. BUSCO analysis revealed that 94.6% of the evaluated genes were complete, comprising 90.8% single‐copy genes and 3.8% duplicated genes (Table S2). After annotation by eggnog‐mapper, 28,271 of the 39,421 proteins (75.40%) with eggNOG_OGs (65.81%), GO (32.47%), COG/KOG (65.81%), and KEGG pathway (19.76%) were assigned using eggNOG annotation results (Figures S1–S3, Table S4).

### 3.2 Genome Evolution Traits of Baiyu Bean

**FIGURE 3:**
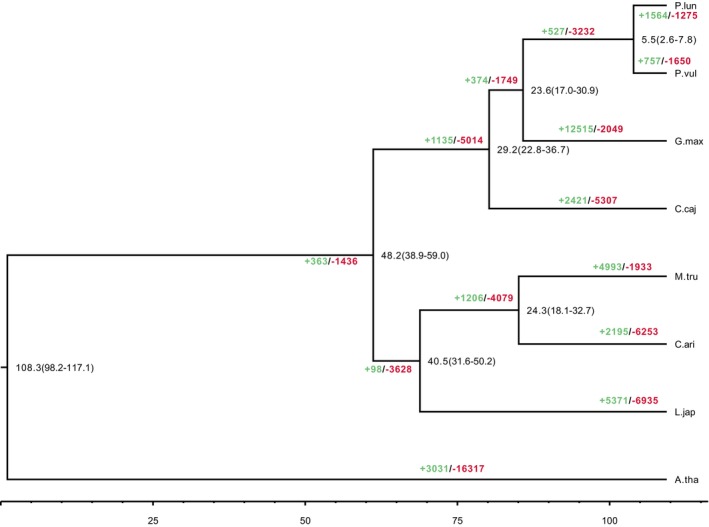
Phylogenetic analysis of baiyu bean with six other legume species. Phylogenetic tree and divergence time of genome‐sequenced legume species with *Arabidopsis thaliana* as an outgroup taxon. The estimated species divergence time is shown at the bottom of the phylogenetic tree. Gene family expansion (green) and contraction (red) are illustrated on the branches and nodes of the tree.

Using OrthoFinder, we detected 642 single‐copy orthologous genes among the 7 legume species (baiyu bean, *C. cajan* , *P. vulgaris* , *C. arietinum* , *L. japonicus* , *G. max* , *M. truncatula* ) (Li et al. 2020; Schmutz et al. 2010, 2014; Varshney et al. 2012, 2013; Young et al. 2011) and *A. thaliana* (Arabidopsis Genome Initiative 2000). Subsequently, we examined the phylogenetic relationships between the baiyu bean and six legume species using 642 single‐copy orthologous genes. As expected, the baiyu bean clustered with the other three legume species (*P. vulgaris*, *G. max*, *C. cajan*) (Figure 3). Model species *A. thaliana* was used as an outgroup. *P. vulgaris* formed a sister lineage to the baiyu bean. Further phylogenetic analysis revealed that the baiyu bean diverged from the common bean at approximately 2.6–7.8 Mya and from soybean at approximately 17.0–30.9 Mya (Figure 3).

Gene family expansion and contraction were examined using CAFÉ (Figure 3). In the baiyu bean genome, 1564 and 1275 genes were expanded and contracted, respectively. Following enrichment analysis, the identified expansion genes were found to be associated with 27 pathways in the Kyoto Encyclopedia of Genes and Genomes (KEGG), including the oxidative pathway, oxidative phosphorylation, photosynthesis, metabolic pathways, carbon metabolism, plant hormone signal transduction, and sesquiterpenoid and triterpenoid biosynthesis. In addition, we detected that these expansion genes were enriched in 199 GO terms, including signal transduction, meristem maintenance, regulation of root meristem growth, response to auxin, iron ion binding, polysaccharide binding, and response to insect (Table S5).

**FIGURE 4:**
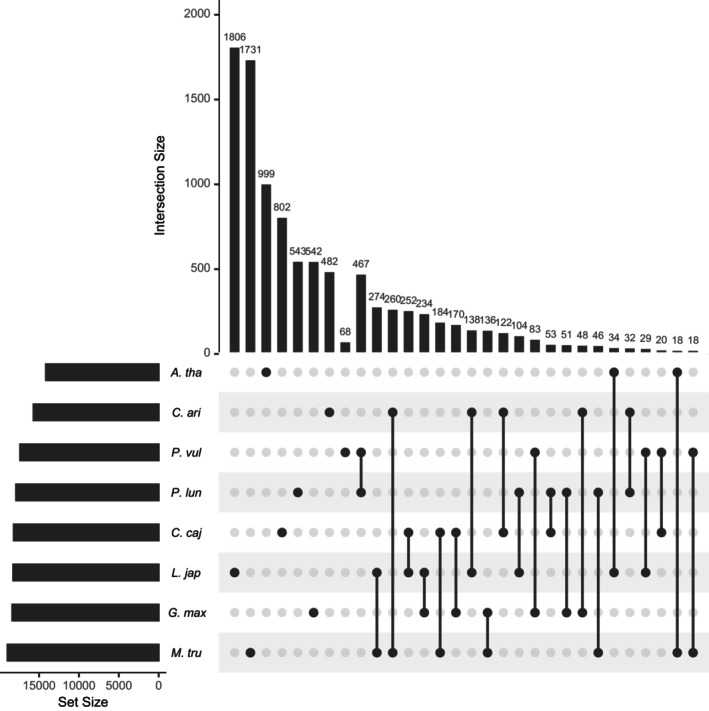
Upset plot of shared and unique gene families in the genomes of *Phaseolus lunatus* , *Cicer arietinum* , *Phaseolus vulgaris* , *Cajanus cajan* , *Lotus japonicus*, *Glycine max* , *Medicago truncatula* , and *Arabidopsis thaliana*.

A total of 543 genes were found to be unique to baiyu bean (Figure 4). KEGG analyses revealed that baiyu bean‐specific genes were notably enriched in 13 terms, including carbon metabolism, other glycan degradation, SNARE interactions in vesicular transport, and metabolic pathways (Table S9). Gene Ontology (GO) analyses indicated that these specific genes were significantly enriched in 186 GO terms, including positive regulation of transcription by RNA polymerase II, embryo development ending in seed dormancy, ADP binding, cytosol, cytoplasm, Cul4‐RING E3 ubiquitin ligase complex, and rRNA processing (Table S6).

Nucleotide diversity at the fourfold degenerate third‐codon transversion site (4DTv) in the baiyu bean genome displayed a distinct peak (4DTv∼0.25) (Figure S4), aligned with the whole‐genome duplication (WGD) event in Papilionoideae (32, 33). The recent whole‐genome duplication peak (4DTv∼0.056) observed in *G. max* was not detected in our analysis, indicating that the baiyu bean does not exhibit this glycine‐specific event, consistent with the majority of sequenced legumes.

We performed an adaptive evolution analysis between two species, *P. lunatus* and *P. vulgaris* . Using Reciprocal Blast, we identified 21,013 gene ortholog pairs between these two species, and further analysis identified 61 genes under adaptive evolution (Table S7), which were enriched in two GO terms: the regulation of secondary cell wall biogenesis and cell–cell signaling involved in cell fate commitment (Table S8). These genes were not significantly enriched in any KEGG pathway.

Positively selected genes in the *P. lunatus* branch were identified using the PosiGene pipeline (Table S9). Five genes were identified as having undergone adaptive evolution: PHALU_013845, PHALU_004007, PHALU_023493, PHALU_019288, and PHALU_010732. Following KEGG enrichment analysis, five genes were identified as enriched in three KEGG pathways: ubiquitin‐mediated proteolysis, oxidative phosphorylation, and starch and sucrose metabolism (Table S10). GO enrichment analysis indicated that these five genes were associated with 26 GO terms, including metaphase/anaphase transition of the mitotic cell cycle, UV protection, positive regulation of the mitotic metaphase/anaphase transition, and early endosome, among others (Table S10).

Furthermore, an adaptive evolution analysis of the branch was performed, including *P. lunatus* and *P. vulgaris*. Eight adaptively evolved genes were detected: PHALU_007406, PHALU_013845, PHALU_019288, PHALU_023493, PHALU_010732, PHALU_025490, PHALU_008196, and PHALU_019458. All eight genes were enriched in four KEGG pathways, including oxidative phosphorylation, protein export, metabolic pathways, and photosynthesis (Table S10). These genes were enriched for 56 GO terms (Table S10). After warm‐season species (phaseoloid/millettioids: four representative species, including *P. lunatus* , *P. vulgaris* , *G. max*, and *C. cajan* ) were set as forebranch, a total of 18 adaptive genes were detected; these genes were enriched in 10 KEGG pathways, including peroxisome, soquinoline alkaloid biosynthesis, ABC transporters, and phenylalanine metabolism, among others (Table S10). These 18 genes were enriched in 81 GO terms, including cytosol, plastid, chloroplast envelope, and proteolysis, among others (Table S10).

### 3.3 Genomic SVs Between Baiyu Bean and G27455

Structural variations (SVs) between the baiyu bean and G27455 genomes were analyzed. Compared with G27455, we identified 5549 SVs, comprising 1553 deletions, 21 duplications, 589 insertions, and four inversions (Table S11). Most SVs were identified in non‐coding regions, with 31.61% located in the intergenic regions, 28.97% in the downstream regions, and 27.03% in the upstream regions. Of the SVs, 1.763% were located in exon regions (Table S12), potentially influencing gene function and contributing to phenotypic divergence between the two cultivars. The highest variant rate was observed on chromosome Pl02 (Table S13). A total of 333 genes, representing 2.338%, were classified as high‐impact structural variant genes (Table S14). These 333 genes were enriched in other glycan degradation pathways after KEGG enrichment. After GO enrichment, these genes were enriched in defense responses to other organisms, exocyst, exocytosis, protein binding, cytoplasm, plasma membrane, and cytosol (Table S15).

## 4 Discussion

In this study, we present chromosome‐scale assembly and comprehensive genomic analysis of the lima bean cultivar baiyu bean. By integrating long‐read nanopore sequencing, Illumina short‐read sequencing, and Hi‐C scaffolding, we generated a highly contiguous genome assembly with 11 pseudomolecules and a total size of 547.93 Mb. The baiyu bean and G27455 genomes both have approximately 41% repetitive content, with LTRs constituting 32.36% and 32.1% of their respective genomes (Garcia et al. 2021). Although the LTR contents were nearly identical, slight differences could reflect variations in transposable element types or genomic distribution. The higher proportion of LTRs in baiyu bean suggests potential differences in transposable element activity or genomic organization, warranting further exploration of their evolutionary role.

In this study, comparative genomic analyses with seven sequenced legumes—*P. vulgaris, C. cajan, G. max, C. arietinum, L. japonicus*, and *M. truncatula* —offer valuable insights to help identify candidates of species‐specific genes, genes involved in adaptive evolution, and to clarify their biological functions (Schmutz et al. 2014; Varshney et al. 2012). This will lead to the evolutionary history, diversification, and habitat adaptation of the family Fabaceae (Chae et al. 2014). The functions of these genes can be evaluated using various in vivo and in vitro gene functional research techniques to clarify their physiological functional characteristics.

Baiyu bean is an excellent lima bean variety cultivated in Jiangxi, Zhejiang, Fujian, and other provinces in China (Xu and Shen 2020). Through genome comparison, we detected 333 SV‐high‐impact genes between G27455 (Garcia et al. 2021) and the baiyu bean (Table S14). GO enrichment results suggested that these SVs may contribute to the differences in disease or pest resistance and developmental traits between these two lima bean cultivars (Table S15). Among these, PHALU_011724‐RA, PHALU_011725‐RA, and PHALU_011727‐RA were CYP82C family members. PHALU_011724‐RA and PHALU_011727‐RA are homologous to *A. thaliana* AT4G31940.1, a member of theCYP82C4 subfamily, which encodes the cytochrome P450 enzyme CYP82C involved in the early iron deficiency response. CYP82C4 hydroxylates fraxetin to form sideretin, both of which are catecholic coumarins secreted into the rhizosphere under low‐iron conditions to mobilize iron from insoluble Fe (III) pools (Paffrath et al. 2024). In addition, PHALU_024116‐RA is homologous to *A. thaliana* AT5G02500.1, encoding a heat shock protein 70 family member. Hsc70‐1 regulates Hsp101 expression through transcription factors HsfA1d, HsfA1e, and HsfA2. The hsc70‐1 mutant showed enhanced thermotolerance, with elevated expression of Hsp101 and other heat shock genes (Berka et al. 2022). The SVs present in these genes may contribute to the differences in stress tolerance between the two varieties, particularly in their responses to both biotic and abiotic stressors. This finding provides valuable insights into the mechanisms underlying the exceptional resistance of baiyu bean to these environmental and biological challenges. A foundation for future genetic development of this cultivar is provided by the high‐quality reference genome of the baiyu bean assembled in this study and the comparative genomic information of the baiyu bean and G27455 collected in this investigation.

Currently, the lima bean genome G27455 and the Bridgeton variety have been reported (Garcia et al. 2021; Wisser et al. 2021), with Bridgeton being a commercially available variety derived from Mesoamerica (Wisser et al. 2021). Here, we report on the local variety of baiyu bean, which has a long history of cultivation in China and shows good adaptability to the middle and lower reaches of the Yangtze River, particularly in areas, such as Jiangxi Province (Xu and Shen 2020). The publication of this genomic data also facilitates the analysis and understanding of genetic variation among different lima bean varieties (Lozano‐Arce et al. 2023). Genome sequencing of superior crop varieties has been performed for crops, such as rice, alfalfa, soybean, and wheat (Jain et al. 2019; Jia et al. 2023; Long et al. 2022; Zhang et al. 2024), promoting the exploration of various excellent genes for disease resistance and agronomic traits. The availability of this genome will help us understand the molecular genetic basis behind the excellent traits of this variety.

In the future, it will be necessary to combine proteomics, transcriptomics, metabolomics, and other multi‐omics approaches to further elucidate the genes responsible for resistance to pests and diseases in baiyu bean, thereby providing a foundation for its genetic improvement. With the publication of genomes from different varieties of lima bean, it is possible to construct a pan‐genome of lima bean, which will further promote the study of genomic features and evolutionary characteristics of lima bean. Such research has already been conducted on various crops, including *Oryza sativa* (Asian rice), *Solanum lycopersicum* (tomato), and *G. max* (soybean) (Della Coletta et al. 2021). The construction of the pan‐genome will expand our understanding of genome content variation among lima bean individuals. In addition, with the development of sequencing technology and telomere‐to‐telomere genome assembly strategies (Garg et al. 2024), it is expected that the telomere‐to‐telomere genome of lima bean will be further assembled, providing strong support for molecular genetic research on lima bean.

## 5 Conclusion

A high‐quality genome of baiyu bean, a local variety of lima beans in China, was obtained in the current study. Using this genomic data, we analyzed the evolutionary characteristics of *P. lunatus* with other legume species. The specific genes and adaptive evolutionary genes of *P. lunatus* were identified. The high‐quality genome obtained will facilitate a deeper understanding of the molecular evolutionary characteristics of *P. lunatus* and promote the development of functional genomics for this species. The current study promotes the exploration of traits and resistance genes of this excellent local variety, facilitating the advancement of genetic improvement in lima bean.

## Author Contributions

**Fengqi Li:** funding acquisition (equal), investigation (equal), writing – original draft (equal). **Junfang Liu:** data curation (equal), software (equal), validation (equal), visualization (equal). **Youssef Dewer:** conceptualization (equal), formal analysis (equal), investigation (equal), validation (equal), writing – review and editing (equal). **Muhammad Haseeb Ahsan:** formal analysis (equal), writing – review and editing (equal). **Chunyan Wu:** conceptualization (equal), formal analysis (equal), resources (equal), writing – review and editing (equal).

## Ethics Statement

The authors have nothing to report.

## Conflicts of Interest

The authors declare no conflicts of interest.

## Supporting information


Figures S1–S4

Tables S1–S15

## Data Availability

The final assembly has been deposited in the NCBI GenBank repository, with the accession number JBJNVO000000000. The raw sequencing data required for de novo genome assembly can be found in the NCBI Sequence Read Archive (SRA) under the accession number: PRJNA731337.
