# Supplementary material for: The Genome of the Lima Bean Variety Baiyu Bean Highlights Its Evolutionary Characteristics
Source: Ecol Evol. 2025 Feb 28;15(3):e71027. doi: 10.1002/ece3.71027 (PMC11868737; doi:10.1002/ece3.71027)

Figure legends

Figure S1. GO Classification of Baiyu bean genes according to the categories of Biological process, Molecular function and Cellular component.

Figure S2. COG/KOG annotations of Baiyu bean proteins. A total of 39,421 predicted proteins have a COG/KOG classification among the 24 categories.

Figure S3. KEGG annotation of Baiyu bean proteins.

Figure S4. Distributions of 4DTv distance within *P.lunatus*, *P.vulgaris*, and *G.max*.

Figure S1.


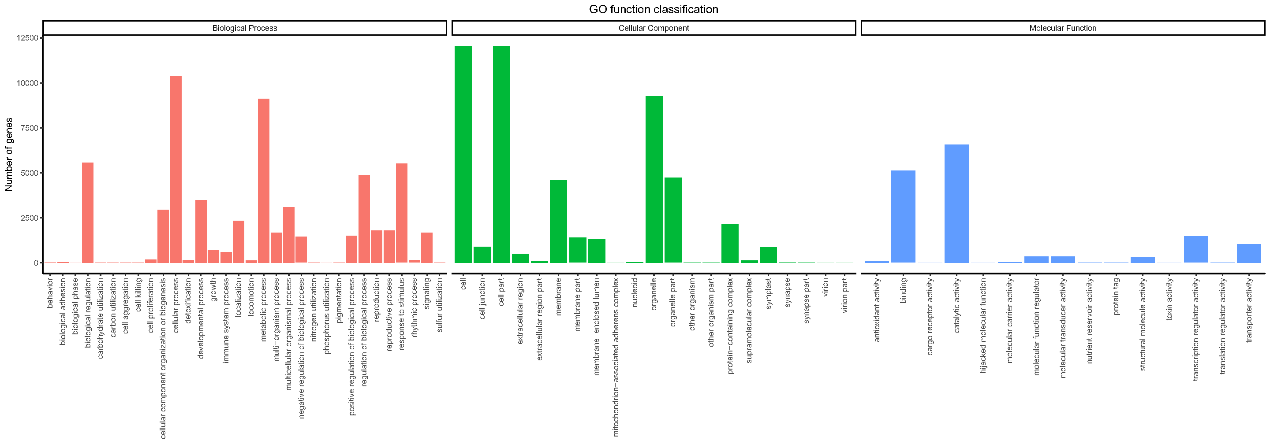


Figure S2.


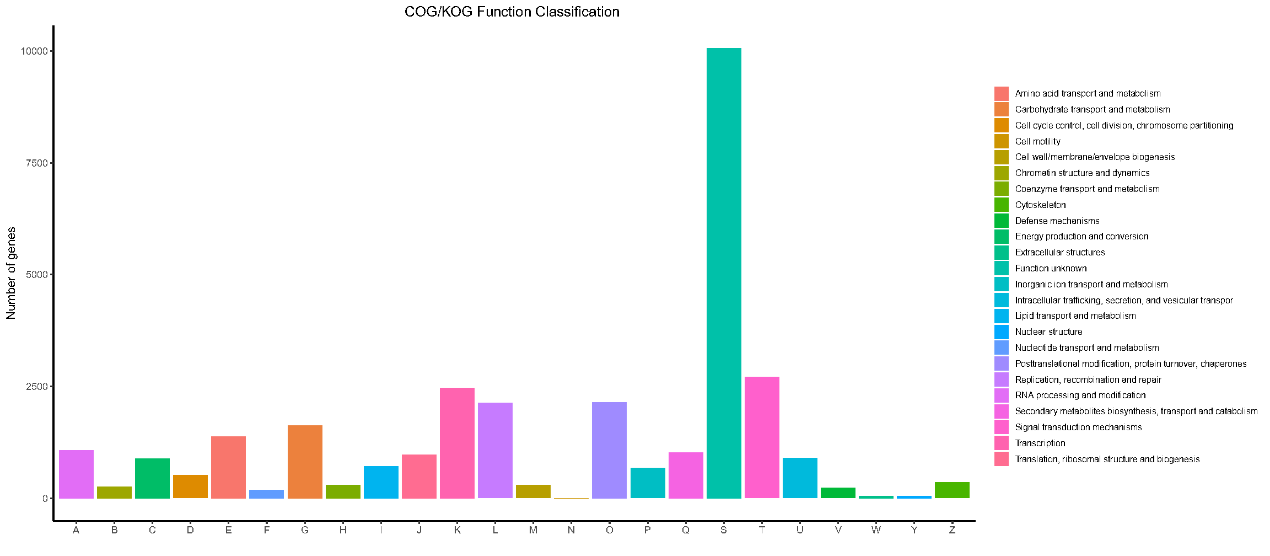


Figure S3.


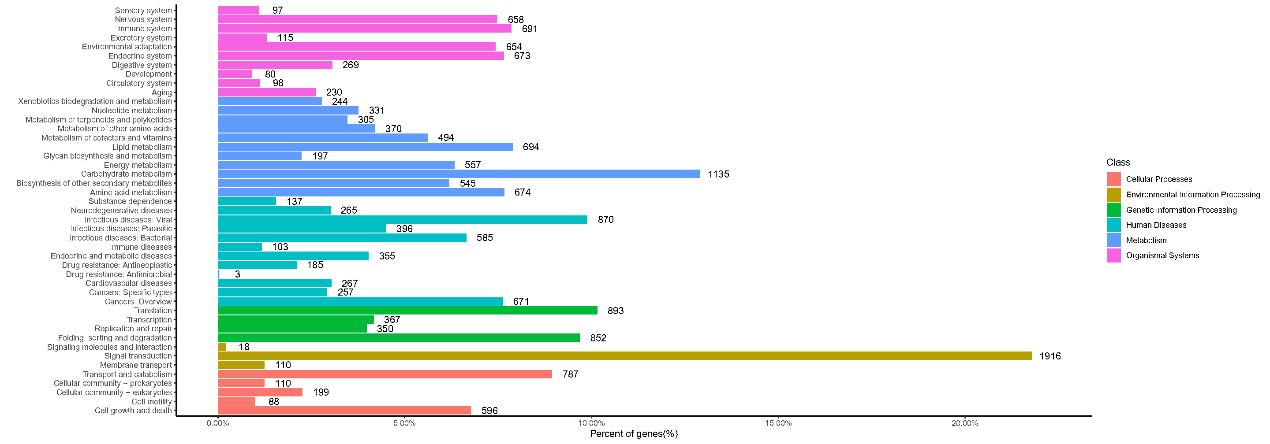


Figure S4.


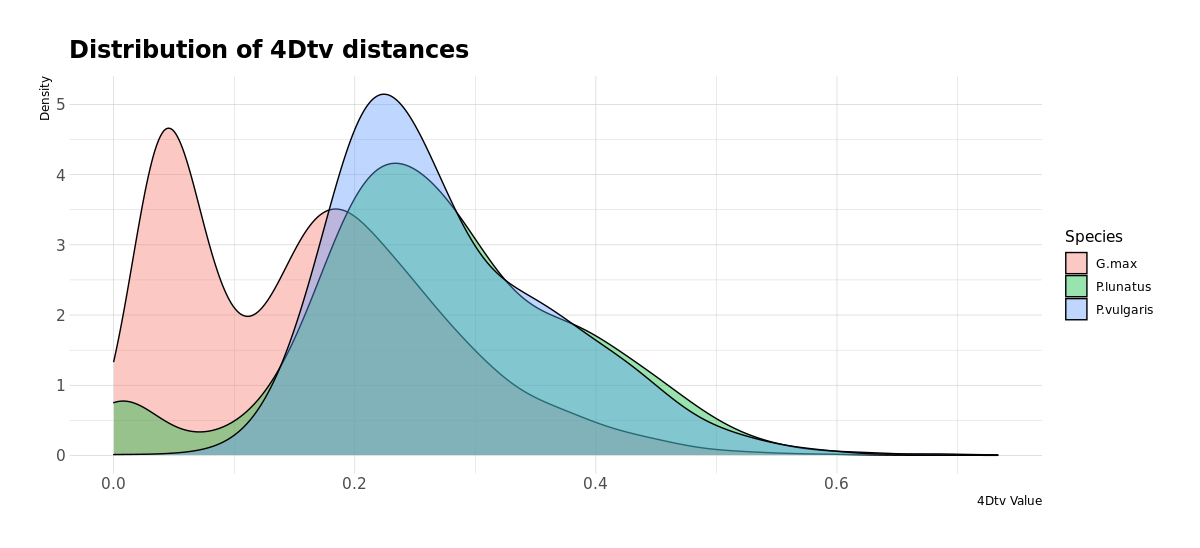

Supplement: Supplementary file 1 — Figures S1–S4 [file ECE3-15-e71027-s001.docx]
